# Some Remarks upon Syphilitic Manifestations in the Larynx*Read by title before the Southern Section of the American Otological, Rhinological and Laryngological Association, Atlanta, March 28.

**Published:** 1898-06

**Authors:** Paul Turner Vaughan

**Affiliations:** Hot Springs, Arkansas


### SOME REMARKS UPON SYPHILITIC MANIFESTATIONS IN THE LARYNX. *

By PAUL TURNER VAUGHAN, M.D,
Hot Springs, Arkansas.

Coming from a health resort where syphilis in its various manifestations is seen constantly, and where innumerable opportunities are presented for studying this disease, I have taken for my subject of discussion to-day Laryngeal Syphilis.

Hot Springs, on account of its wonderfully beneficial thermal waters, has become the city toward which, yearly, thousands of invalids suffering with syphilis travel, and in no other city that I am familiar with, except perhaps Vienna, is it possible to study this disease in so many interesting and varied phases.

I shall lay before you to-day some facts coming from several years of close study and observation upon this subject, and while they are not new facts to such a body of physicians as this is, still they are facts which will bear repetition, for in dealing with this disease we are dealing with one insidious in its approaches and far-reaching in its ravages upon the human body, if its course be not checked early by appropriate treatment.

Syphilitic manifestations in the larynx are met with rather frequently, and, like the manifestations of syphilis in any other portion of the body, may be divided arbitrarily into three stages, primary, secondary and tertiary.

I do not think that the division of syphilitic manifestations into secondary and tertiary is a good division, because we constantly see secondary manifestations occurring in what is regarded the tertiary stage, and *vice versa*, tertiary manifestations often occurring in a few months after the initial lesion, and in the period which some would designate secondary.

I have often seen mucous patches occur in the throat fifteen

---

* Read by title before the Southern Section of the American Otological, Rhinological and Laryngological Association, Atlanta, March 28.

years after the initial lesion, and conversely, I have seen gummata occur within six months after the primary sore.

However, in lieu of some better classification, I shall speak of the symptoms as being primary, secondary and tertiary.

Personally, I have never seen a primary syphilitic sore situated in the larynx, and in my reading upon the subject have only found reference to one such case, but the erythema, mucous patch, ulcerative process and gumma, followed later by breaking down of the gummy tumor, and the subsequent formation of cicatricial tissue, and the occurrence of stenosis of the larynx in varying degrees, are, with the possible exception of the mucous patch, rather common pictures to the laryngologist.

Mucous patches are considered a rather characteristic symptom of syphilis, and although of rare occurrence, they do sometimes exist in the larynx.

When found present in this locality they appear by preference to locate upon the upper surface and free margin of the epiglottis on the false vocal cords, and sometimes are found on the inter-arytenoid commissure.

A quite marked symptom in a great many cases of syphilis in its early stage is the appearance of laryngeal catarrh, and only in one particular does this differ from an ordinary catarrh, the last named variety being usually relieved by topical applications while they are only of limited value in the specific type, one being compelled to resort to antisyphilitic treatment in order to relieve the condition.

The erythema observed in the larynx is not so marked and is not so characteristic as is the erythema so often found present in syphilis of the larynx, but it is sufficiently noticeable, particularly when associated with alternating patches of red and white situated upon the vocal cords to lead one to suspect its specific origin.

Pain is generally absent, and the condition may last a long time showing no tendency to pass into one of the graver manifestations of syphilis. There are no markedly uncomfortable symptoms produced by the erythema unless the vocal cords become involved. If the vocal cords become involved there may result much impairment of the voice; in fact, I have observed some cases in which the voice was completely lost.

Probably the most common manifestation of syphilis in the tertiary form in the larynx is ulceration. This is preceded by an infiltration of the mucous membrane with inflammatory products and is usually diffuse in character.

Gummata are commonly situated near the posterioı commissure, upon the arytenoid cartilages and upon the epiglottis. At first they are small, of the same color as the contiguous mucous membrane and often passed unnoticed for some time, but gradually they enlarge, soften, a small yellow spot appears in the center of each gumma, and finally a destructive ulcerative process begins.

This ulcerative process may be either superficial or deep, the superficial variety occurring in what is generally called the secondary stage. From the cases coming under my observation I am inclined to the belief that both varieties of ulceration are the result of the disintegration of gummy tumors, the superficial variety occurring when the tumor is situated just beneath the mucous membrane, and the deep variety occurring when the tumor is more deeply situated; hence, I speak of both varieties as occurring in the tertiary stage of the disease. Ulceration, when once established, progresses very rapidly, and frequently large areas are completely destroyed.

When ulceration occurs on the epiglottis, entire destruction of the valve may take place before the patient can be brought under the influence of medicine. When much tissue has been destroyed there will, of course, after healing begins, be a formation of cicatrices with resulting stenosis, and sometimes great distortion of the larynx.

When this occurs the voice is permanently much altered in character.

On account of the clinical history of the case there is generally no trouble in making a diagnosis of laryngeal syphilis, but sometimes a case is met with in which the diagnosis is exceedingly difficult to make.

Syphilitic ulcers in the larynx have sharply defined borders, are excavated, have a dirty, purulent slough upon their floors, and are surrounded by angry looking, very much inflamed mucous membrane, and in addition there is comparative absence of pain.

Tuberculous ulcers present an entirely different clinical picture, having ragged, illy-defined borders, are superficially situated, are rather grayish looking in appearance, are not surrounded by inflamed mucous membrane, and the pain is severe and almost constant. In doubtful cases an examination of the lungs and of the sputum will usually aid in establishing a diagnosis.

The most difficult cases I have had any experience with in establishing a diagnosis have been in cases of malignant disease of the larynx. In carcinoma, after ulceration begins, the differential diagnosis is very hard to make, and I confess it has been my experience that only treatment with the iodide will establish a diagnosis.

With lupus of the larynx I have had no experience, having only observed one case, and consequently cannot speak from personal observation upon this point, but it is said that lupus is rarely, if ever, primarily seen in the larynx.

Before proceeding to the subject of treatment I shall refer to several bodily conditions which influence seriously both the course and treatment of this disease.

Anemia and general malnutrition, when present, necessarily allow the syphilitic infection to become general earlier, and to pass through its various stages in a much shorter period than when the patient is in good bodily condition.

One of the most serious complications possible in a patient suffering with syphilis is for the patient to develop tuberculosis.

A patient in whom syphilis is present, owing to the necessarily weakened condition of his system and consequently its inability to resist infection, offers a peculiarly fertile soil for the active and rapid development of tuberculosis.

Another condition which exerts an unfavorable action upon the course of syphilis is the presence of a chronic nephritis.

Both mercury and the iodide are to a great extent eliminated by the kidneys, and when these organs are diseased the administration of both drugs, in sufficient quantity to accomplish benefit, is seriously interfered with.

Arterial disease, or arterio-sclerosis, is another condition occurring in elderly people, which may influence the course of the disease, especially as the blood-vessels seem to be particular objects of attack by the syphilitic virus.

Malaria appears also, when present, to cause an increase in the severity of syphilitic symptoms, especially an increase in the neuralgic symptoms.

Now the treatment of syphilis when present in the larynx varies little from the treatment of syphilis in any other portion of the body, except that skill in making applications to the larynx is essential.

It is generally considered, I know, that to wait the development of secondary symptoms, and then begin constitutional treatment, is the better course to pursue, but it appears to me that more favorable results are obtained when the patient is put upon some one of the mercurial salts as soon as the diagnosis of syphilitic infection is positively decided upon. Should a primary sore be encountered in the larynx, the treatment of it would not differ materially from one found in any other portion of the body.

It should be cleaned, and dusted with some dried antiseptic powder, as iodol, europhen, calomel, or with iodoform. This latter powder is most efficient in syphilitic sores, but at the same time is very disagreeable to the patient.

Upon the appearance of secondary symptoms, either inunctions with mercurial ointment or the internal administration of some salt of mercury will cause subsidence of the symptoms. The mucous patch, when found in the larynx, is, as elsewhere in the body, resistant to treatment, and is very likely to recur, but daily touching with tincture of iodine will usually cause its disappearance. Catarrh of specific origin requires, in addition to constitutional treatment, the administration of ferruginous preparations, and the spraying of the naso-pharynx with mild antiseptic and alkaline solutions.

Upon the appearance of tertiary manifestations, the administration of the iodid of potassium or sodium is indicated, beginning with small doses of from ten to twenty grains three times a day, and increasing the dose several grains daily until the progress of the disease is checked.

In Hot Springs larger doses of the iodide can be given and will be tolerated better, on account of the hot baths and their diaphoretic action, than can apparently, with comfort to the patient, be given elsewhere.

The baths are of great benefit in that they keep the skin in a thoroughly healthy condition, and thus aid wonderfully in eliminating poisonous principles from the body.

In some obstinate cases of severe ulceration, and also in those cases where impending disintegration of gummata is present, and a quick effect is desired from medicine, it is best to combine mercurial inunction with the iodide. With treatment by means of hypodermic injections of the insoluble salts of mercury, I have had no experience, and hence cannot speak on this subject knowingly; but in the hands of other men I have seen consequences follow this mode of treatment which could have been obviated by employing the inunction method.

When cicatricial stenosis ensues from ulceration in the larynx, the use of instruments for dilating the stricture becomes necessary. Probably the best instrument for this purpose is a three-bladed dilator described by Schroetter.

When the larynx is not too much distorted, an intubation tube can be used with advantage. The use of iodid of potassium is, I think, uncalled for in these cases, producing a variety of iodic laryngitis which is very distressing to the patient.

